# From empirical vaccinology to predictive systems-based vaccine design: multi-omics integration, artificial intelligence, and global equity challenges

**DOI:** 10.3389/fsysb.2026.1819469

**Published:** 2026-04-30

**Authors:** Nicole Simone De Lima Coelho, Viviana Simone De Lima Coelho

**Affiliations:** 1 School of Medicine, Faculdade de Medicina de Marília (FAMEMA), Marília, São Paulo, Brazil; 2 School of Psychology, Universidade de Marília (UNIMAR), Marília, São Paulo, Brazil

**Keywords:** immune response, immunology, multiomics, vaccines, vaccinology

## Abstract

Although vaccination remains one of the most impactful interventions in contemporary public health, with consistent and widely documented reductions in morbidity and mortality, the traditional empirical development of vaccines—largely based on trial-and-error strategies—still reflects an incomplete understanding of the complexity underlying immune responses. With the emergence of systems immunology, supported by multi-omics technologies, mathematical modeling, and computational tools, vaccinology has progressively incorporated the integration of multiple biological layers, addressing critical mechanistic gaps and mitigating limitations of the classical model, thereby transitioning from an empirical framework toward a predictive and integrative paradigm. In this context, the present review critically examines how systems immunology contributes to rational vaccine design by exploring its technological foundations—particularly omics approaches—discussing strategies for data integration, analyzing translational implications, and incorporating considerations related to Artificial Intelligence (AI), regulatory governance, ethics, and global equity. Within this evolving landscape, systems vaccinology has demonstrated promising results and optimistic perspectives, particularly regarding predictive capacity, immunological stratification, vaccine personalization, and potential epidemiological impact. At the same time, challenges including reproducibility concerns, risk of overfitting, the distinction between multi-omic correlation and functional causality, the need for longitudinal and experimental validation, algorithmic bias, excessive reliance on computational models, and regulatory barriers to the approval of data-driven vaccines represent important limitations of this approach. Taken together, these considerations indicate that systems immunology constitutes not merely a technological refinement but a genuine paradigm shift, redefining vaccine development as a predictive, iterative, and integrative process that must be scientifically validated and ethically contextualized, with profound implications for global public health.

## 1 Introduction

Vaccination has become one of the most impactful public health interventions worldwide (Raja and Ashwinth Jothy, 2024). The importance of vaccines in global health is undeniable, as the advent of immunization has prevented countless complications and deaths. For example, between 2020 and 2024, it is estimated that more than 2.5 million deaths from COVID-19 were averted due to vaccination (Ioannidis et al., 2025).

The immune system operates as a highly interconnected network involving cellular, molecular, and signaling components, whose interactions determine the quality and magnitude of protective responses (Sabir and Jan, 2025). Historically, vaccine development relied predominantly on empirical approaches, which proved highly successful against many acute infectious diseases.

However, the emergence of pathogens characterized by immune evasion, antigenic variability, and complex host–pathogen interactions has exposed the conceptual limitations of classical vaccinology (Pollard and Bijker, 2021). Insufficient mechanistic understanding of immune development, host genetic heterogeneity, environmental influences, and safety constraints further complicates the rational design of broadly protective vaccines. Diseases such as tuberculosis, malaria, HIV infection, hepatitis C, and emerging coronaviruses exemplify how biological complexity challenges traditional empirical paradigms (Kennedy et al., 2020).

Systems immunology reframes immune responses as dynamic, multi-layered biological networks, integrating multi-omics profiling with computational and mathematical modeling to identify molecular signatures and pathways associated with protective immunity (Alfonso-González et al., 2025). Importantly, its translational relevance depends not solely on high-throughput data generation, but on biologically grounded interpretation of complex datasets within coherent immunological frameworks. In the context of vaccine development, such systems-based strategies support rational vaccine design by informing antigen selection, delivery platforms, and adjuvant optimization through mechanistic insights into immune activation and regulation (Schijns et al., 2021).

These advances have given rise to strategies such as vaccinomics, which leverages omics data to characterize immune responses, reverse vaccinology, which identifies immunogenic targets directly from genomic information, and structure-guided vaccine design. The practical impact of this paradigm is exemplified by the development of the serogroup B meningococcal vaccine, in which genome-based antigen discovery enabled the identification of multiple surface-exposed protein candidates beyond those detected through conventional methods (Gasparini et al., 2015; Maiden, 2018; Kennedy et al., 2020). This case illustrates how systems-based and genome-guided strategies can enhance the efficiency and breadth of antigen selection compared with traditional empirical approaches.

**FIGURE 1:**
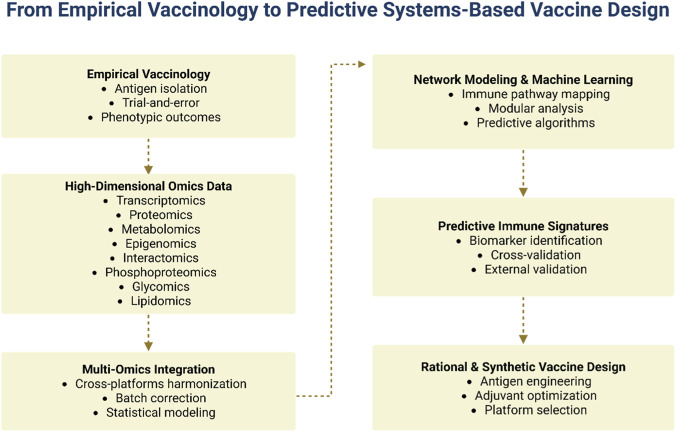
Conceptual transition from empirical to systems-based predictive vaccine design. The figure represents the shift from traditional empirical vaccinology toward a data-driven, systems-level approach. The visual metaphor of a fragmented and evolving path illustrates the transition from linear, trial-and-error strategies to a more complex and integrated framework supported by multi-omics data generation, computational modeling, and cross-platform data integration. This transformation enables the identification of predictive immune signatures and supports rational vaccine design through informed decision-making in antigen selection, adjuvant optimization, and platform development (created by the author with BioRender.com).

Building on this framework, this article examines how systems immunology, supported by multi-omics integration, contributes to rational vaccine design within the broader landscape of synthetic immunology. We argue that the transition from empirical to systems vaccinology represents a multilayered and conceptually transformative process, integrating high-dimensional data generation, computational modeling, and rational vaccine engineering. This integrative and conceptually transformative transition is schematically summarized in Figure 1.

## 2 Methodology

This study is a narrative review examining the transition from empirical vaccinology to systems-based rational vaccine design, integrating conceptual, technological, translational, ethical, and global health perspectives. The literature search was conducted exclusively in the PubMed database, selected for its comprehensive indexing of peer-reviewed biomedical research and its relevance to immunology and vaccinology.

Search terms were applied individually and in combination using Boolean operators (AND, OR), encompassing descriptors related to systems immunology, classical and empirical vaccinology, rational vaccine design, systems vaccinology, multi-omics approaches, data integration, artificial intelligence, machine learning, vaccine biomarkers, and global health. Priority was given to studies published within the last 10 years to capture recent advances, while seminal earlier works were included when necessary for conceptual grounding.

Studies were selected based on relevance to systems-based approaches in vaccine research, including investigations involving multi-omics integration, predictive modeling, biomarker discovery, artificial intelligence applications, and ethical or regulatory dimensions of data-driven vaccinology. Articles outside the scope of systems-oriented vaccine research or lacking methodological clarity were excluded.

The analytical process was interpretative and critical, aiming not only to synthesize technological developments but also to examine methodological limitations, translational challenges, regulatory considerations, and issues of global equity.

## 3 Historical evolution of vaccinology

The concept of immunity dates back to the 14th century, during the emergence of bubonic plague. In the 18th century, experimental approaches advanced with the natural sciences, notably through the inoculation of cowpox material by Edward Jenner in 1796 to prevent smallpox. At that time, immunizing agents were still rudimentary; only in the late 19th century did vaccines begin to be developed in laboratory settings, with attenuation methods introduced by Louis Pasteur and inactivation approaches proposed by Salmon and Smith.

In the 20th century, vaccine research focused on bacterial and viral pathogens, as well as antibody responses and attenuation strategies. By 1929, vaccination had enabled the control of diseases such as smallpox, rabies, typhoid fever, shigellosis, cholera, tetanus, and pertussis (Plotkin, 2014; Soleymani et al., 2022; Montero et al., 2023; Kajal et al., 2024).

Although the empirical approach to vaccine development has been highly effective, important limitations remain, including the need to improve efficacy and address emerging complex diseases. These challenges are partly related to an incomplete mechanistic understanding of the immune system, with vaccine discovery often relying on phenotypic observation and lacking predictive capacity. In this context, systems vaccinology offers a more mechanistic and predictive framework for rational vaccine design (Poland et al., 2011; Sharma et al., 2019).

## 4 Limitations of classical empirical vaccine development

The development of vaccines currently relies on advanced technologies. However, in earlier periods, due to the lack of such resources, vaccine formulation was guided primarily by trial and error, i.e., it was conducted empirically. In this context, the strategies employed included, for example, animal protection studies, passive administration of antibodies against specific antigens, and the culture of microorganisms.

Based on these approaches, classical vaccinology revolutionized public health through the development of immunizers against diseases such as smallpox (1798), rabies (1885), typhoid fever (1896), cholera (1896), bubonic plague (1897), diphtheria toxoid (1923), pertussis (1926), tetanus toxoid (1926), tuberculosis (1927), yellow fever (1935), influenza (1936), rickettsial diseases (1938), among others.

Nevertheless, although numerous breakthroughs were achieved through the empirical approach, it presents important limitations that systems vaccinology seeks to overcome. These include the limited understanding of immune system activation, the identification of critical protective antigens and the immune responses they elicit—including innate immune responses—the personalization of vaccines for specific population groups, and the development of strategies to address the high antigenic variability of certain microorganisms.

Regarding the limited understanding of the immune system, unlike systems vaccinology, empirical vaccinology primarily relies on pathogen cultivation and does not commonly incorporate molecular analysis. As a result, the data obtained tend to be less detailed than those generated by systems vaccinology, which employs high-throughput molecular approaches.

With respect to the identification of critical protective antigens, more recent vaccinology frameworks enable the detection of genetic polymorphisms that may influence gene expression. In this context, this deeper level of analysis is particularly important when considering vaccine personalization, especially for specific population groups. Such individualization becomes a more prolonged process within empirical vaccinology, as similar insights can only be reached indirectly, often after multiple trial-and-error attempts.

Furthermore, in relation to the development of strategies to target highly variable antigens, the identification of surface proteins represents a key factor. However, in empirical vaccinology, this information is also obtained indirectly, depending on observations of infection outcomes and host immune responses. In contrast, in systems vaccinology, such data can be obtained directly, which may provide greater precision in vaccine formulation, allowing immunizers to be designed with specific targets in mind.

In this context, systems immunology has increasingly been applied to support rational vaccine design, aiming to achieve greater efficacy, improved safety, and reduced reactogenicity (Buonaguro et al., 2011; Plotkin, 2014; Zepp, 2016; Oli et al., 2020; Kajal et al., 2024).

## 5 Systems immunology: concept and principles

Traditionally, classical immunological research adopts a reductionist perspective, focusing on isolated cell types and signaling pathways. In contrast, systems immunology is a research approach that explores the relationships among multiple modulatory components of major immune signaling pathways—at the molecular, cellular, and tissue levels—under perturbations such as environmental or genetic factors, based on mathematical models and computational tools. In this sense, it enables a more comprehensive perspective (Eftimie et al., 2016; Villani et al., 2018; Fu et al., 2023).

By operating on multi-omics data, including transcriptomic, proteomic, and metabolomic information, this approach enables the identification of molecular signatures associated with immune activation, regulation, and memory formation. For example, early transcriptional signatures in peripheral blood have been used to predict vaccine immunogenicity, while integrative analyses of cellular and cytokine responses have helped identify correlates of protection and mechanisms of vaccine-induced immunity.

In practical terms, systems immunology can directly inform vaccine development by supporting antigen selection, immune profiling, and adjuvant optimization through integrative multi-omics analyses and computational modeling. These approaches have been applied to next-generation vaccine platforms, such as mRNA, DNA, and nanoparticle-based vaccines, thereby enabling improved control over antigen presentation, immune activation, and response durability, as demonstrated in studies of pathogens including SARS-CoV-2, Epstein–Barr virus, and *Mycobacterium tuberculosis*. From this standpoint, systems immunology facilitates the prediction of biological responses and supports the rational development and refinement of immunotherapies, including vaccines (Eslami et al., 2025).

Although the yellow fever YF-17D vaccine is one of the most effective vaccines ever developed and was originally designed through classical attenuation approaches, it has subsequently been used as a model to analyze immune responses using systems immunology. These analyses have characterized transcriptional, cellular, and cytokine responses—particularly antigen-specific CD8^+^ T cell activity and neutralizing antibodies—which could be predicted with over 90% accuracy by computational tools.

In addition, systems immunology studies using YF-17D have enabled the identification of early molecular signatures associated with protective immunity, contributing to the discovery of potential correlates of protection. This predictive capacity exemplifies the transition from empirical to data-driven vaccinology, in which integrative multi-omics analyses and computational modeling allow not only the characterization but also the anticipation of vaccine-induced immune responses, including their magnitude and durability, thereby supporting more rational and efficient vaccine development (Rappuoli et al., 2024).

Another relevant aspect to consider is that, through this technology, it is possible to understand the interactions between immunology and other systems within the human body, such as the impact of the gut microbiota on vaccine responses, based on differential gene expression profiles and pro-inflammatory transcriptional and cellular responses. For instance, antibiotic-induced perturbations of the gut microbiome have been shown to reduce antibody responses to vaccination, particularly in individuals with low pre-existing immunity, while also promoting systemic inflammatory signatures resembling those observed in aging. These effects are associated with alterations in microbiota-derived metabolites, highlighting a mechanistic link between microbial composition, host metabolism, and immune function. Such findings, supported by integrated transcriptomic, metabolomic, and microbiome analyses, exemplify how systems immunology can uncover complex host–microbiome interactions that directly influence vaccine immunogenicity (Hagan et al., 2019). Therefore, this approach is highly valuable for vaccine development, as it provides a holistic view that integrates multiple biological systems into immune response prediction and enables greater precision throughout the testing phase until completion.

## 6 Multi-omics approaches in systems immunology

**FIGURE 2:**
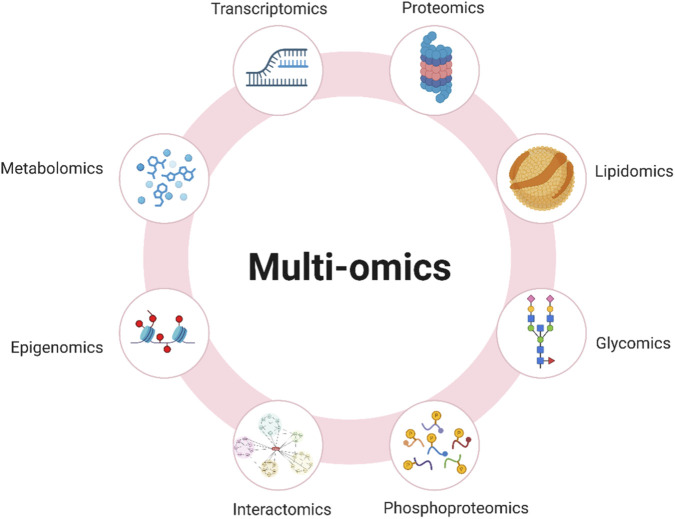
Multi-omics as an integrative framework in systems vaccinology. The figure illustrates the major omics layers—transcriptomics, proteomics, lipidomics, glycomics, phosphoproteomics, interactomics, epigenomics, and metabolomics—that collectively capture different dimensions of biological regulation. These complementary datasets provide a multi-layered view of molecular and cellular processes, enabling integrative analyses that support mechanistic understanding and predictive modeling of immune responses. When combined, these approaches form the basis of systems-level strategies for rational vaccine design (created by the author with BioRender.com).

Omics technologies generate molecular-, cellular-, and tissue-level datasets that interrogate distinct yet interconnected dimensions of immune biology. Each omics approach captures specific biological information; however, their true conceptual strength lies in the integration of these complementary layers into coherent systems-level models (Babu and Snyder, 2023). As illustrated in Figure 2, these technologies should not be interpreted as isolated analytical domains, but rather as functional strata that collectively contribute to rational vaccine design.

### 6.1 Early immune activation and predictive signatures

#### 6.1.1 Transcriptomics and phosphoproteomics — applied emphasis

Transcriptomic technologies encompass multiple methodological approaches that differ in spatial resolution, gene detection capacity, and transcriptome coverage, including sequencing-based, probe-based, image-based, and image-guided single-cell RNA sequencing techniques (Chen et al., 2023). These approaches allow high-resolution analysis of gene expression dynamics at cellular or subcellular levels and have become central to the identification of vaccine-induced immune signatures.

For example, in individuals receiving the trivalent seasonal influenza vaccine, transcriptomic profiling of peripheral blood revealed downregulation of the histone acetyltransferases CREBBP/CBP and KAT6A at 1-, 3-, and 7-days post-vaccination. When integrated with epigenomic data, these findings indicated a pronounced hypoacetylation state in myeloid cells following immunization (Wimmers et al., 2021). This study exemplifies how transcriptional data gain interpretative depth when analyzed alongside regulatory layers.

Phosphoproteomics complements transcriptomics by profiling phosphorylated peptides *in vivo* using high-throughput mass spectrometry (Nilsson, 2012; Stasyk and Huber, 2012; Liu et al., 2013). Because protein phosphorylation modulates activity, localization, and protein–protein interactions, phosphoproteomic analyses provide insight into dynamic signaling events and early activation pathways.

Despite their predictive potential, important limitations must be acknowledged. Transcriptomics measures gene expression rather than direct protein function and is highly dependent on the timing of sample collection. Phosphoproteomic analyses may face technical complexity and limited reproducibility due to the transient nature of phosphorylation events. Furthermore, predictive signatures derived from specific cohorts may not be generalizable across populations with different genetic backgrounds, immune states, or vaccine platforms (Ramos, 2025). Careful analytical interpretation is therefore required to avoid conflating correlation with causality.

### 6.2 Functional effectors and network architecture

#### 6.2.1 Proteomics and interactomics — critical emphasis

Proteomics, primarily based on mass spectrometry, enables classification and quantification of proteins according to their biochemical properties (Chen et al., 2020) and provides a closer approximation to functional immune output than transcriptomics alone. In vaccine-related research, proteomic analyses have been applied to characterize vaccine-induced immune responses and identify molecular correlates of protection. For example, peptide microarray–based approaches can be used to identify viral peptide expression profiles associated with post-vaccination immune responses. By comparing pre- and post-immunization profiles, these strategies may enable the prediction of individual immune responsiveness, allowing the identification of low responders who could benefit from additional vaccine doses or tailored immunization strategies (Galassie and Link, 2015).

Although proteomic approaches have also been applied to investigate host–pathogen interactions, including Influenza A virus infection, these studies provide complementary insights into immune mechanisms that can inform vaccine design (Haas et al., 2023).

Interactomics extends this functional dimension by mapping intra- and intermolecular protein interactions. Within systems biology frameworks, immune responses are understood as emergent properties of complex and dynamic molecular networks, in which proteins act not in isolation but as components of highly coordinated interaction systems that regulate signal transduction and cellular function (Bakhtina et al., 2024). Physical interactions at cellular membranes are central to immune processes such as dendritic cell–mediated T cell activation and CD4^+^ T cell–dependent B cell maturation. From a systems vaccinology perspective, these interaction networks are essential for understanding how innate immune sensing is translated into adaptive immune responses, as coordinated signaling between immune cells determines the magnitude, quality, and persistence of vaccine-induced immunity (Elzawahry et al., 2014; Moreno-Pérez and Patarroyo, 2020; Nakandakari-Higa et al., 2024). Network-level analyses may therefore reveal critical nodes within immune response pathways.

However, proteomic analyses may not detect all proteins, particularly those in low abundance, and interactomic networks can become highly complex and difficult to interpret. Moreover, not all detected interactions are functionally relevant. These limitations underscore the need for functional validation and integration with complementary datasets to ensure biological plausibility and translational relevance.

### 6.3 Regulatory programming and immune memory

#### 6.3.1 Epigenomics — conceptual emphasis

Epigenomic technologies investigate chromatin folding, nucleosome positioning, histone tail modifications, and DNA methylation patterns that shape gene expression and influence immune responses (Wang and Chang, 2018). This regulatory layer functions as an intermediary between genetic information and functional immune output.

In vaccinology, epigenetic mechanisms play a central role in shaping vaccine-induced immune responses. Epigenetic reprogramming of innate immune cells following vaccination has been associated with enhanced responsiveness upon secondary stimulation, a phenomenon known as trained immunity (Divangahi et al., 2021). In addition, epigenomic signatures have been linked to variability in vaccine responsiveness, suggesting a role in predicting immunogenicity and long-term protection, considering the diversity of epigenetic pathways, including DNA methylation, histone acetylation, three-dimensional nuclear architecture, and enhancer RNAs (Fanucchi et al., 2021).

These findings highlight the importance of epigenetic regulation in both the magnitude and durability of vaccine-induced immunity. However, epigenetic modifications are often reversible and highly context-dependent, complicating the interpretation of their functional relevance. Establishing direct causality remains challenging, and there is a risk of overinterpreting correlational associations without longitudinal validation.

Thus, epigenomics contributes mechanistic insight into immune variability and memory formation but requires integration with transcriptional and functional data to support robust translational applications.

### 6.4 Metabolic and structural interfaces

#### 6.4.1 Metabolomics, lipidomics, and glycomics — prospective emphasis

Metabolomics measures small molecules with diverse physicochemical properties, including lipids, sugars, nucleotides, amino acids, and amines. The metabolome encompasses compounds derived from endogenous enzymatic activity as well as from diet, medications, microbiota, and environmental exposure (Gerszten and Wang, 2008; Clish, 2015). Because these metabolites are directly linked to cellular processes, they have been regarded as disease indicators.

Immune activation is closely associated with metabolic reprogramming, including glycolysis, the tricarboxylic acid cycle, the pentose phosphate pathway, fatty acid metabolism, and amino acid pathways such as glutamine and tryptophan metabolism (Chou et al., 2022). These metabolic states may influence vaccine responsiveness and offer potential for therapeutic optimization.

Lipidomics, now considered a distinct omics field due to lipid diversity (Wang et al., 2020; Wu et al., 2020), has demonstrated relevance in viral infections. In rotavirus infection, alterations in cellular lipid metabolism, particularly ceramide accumulation, were observed in infected enterocytes (Gaunt et al., 2013; Tao et al., 2024). Given the central role of lipids in membrane structure and signaling, lipidomics is especially relevant for mRNA vaccine platforms employing lipid nanoparticles.

Glycomics encompasses the analysis of glycan-related biomarkers and glycosylation patterns. Glycans located on cell surfaces play roles in signal transduction, adhesion, and pathogen binding, and more than half of human proteins are glycoproteins (An et al., 2009; Peng et al., 2018; Smith et al., 2019). Glycan biosynthesis, unlike protein synthesis, is not template-driven, leading to substantial structural variability. This characteristic contributes to their relevance in host–pathogen interactions and in glycoprotein-based vaccine design, including RNA-based vaccines targeting the SARS-CoV-2 spike protein (Wang et al., 2021).

Despite their translational promise, metabolomic and structural analyses face challenges including environmental variability, methodological heterogeneity, and difficulties in distinguishing causative mechanisms from downstream metabolic consequences. Their integration with upstream transcriptional and proteomic datasets is therefore essential for biologically coherent and clinically applicable modeling.

## 7 Data integration

The integration of omics data enables a holistic perspective that cannot be achieved through individual datasets alone. For instance, it allows the investigation of associations between the composition of the gut microbiota and immune response profiles, which may promote a pro-inflammatory state. Another example is the assessment of the impact of aging on immunity, taking into account age-related alterations in the microbiota, which likewise indicate a tendency toward a pro-inflammatory state. In addition, antibiotic use appears to have the capacity to induce changes that lead to inflammatory responses (Hagan et al., 2019; Chou et al., 2022). Despite challenges related to the standardization of data integration—such as heterogeneity, storage requirements, data size, and format of individual omics datasets—the multi-omics approach remains a valuable strategy for generating insights in the field of synthetic immunology, particularly with regard to vaccine development (Subramanian et al., 2020).

Nevertheless, beyond technical standardization challenges, data integration represents a central conceptual bottleneck in systems vaccinology. The ability to generate large-scale datasets has advanced substantially; however, technological capacity must be accompanied by biological interpretability in order to produce meaningful translational insights.

However, it is important to recognize that each omics layer interrogates a distinct biological dimension—such as RNA, proteins, lipids, among others—and that the generation of large-scale datasets is fundamentally different from understanding the underlying mechanisms involved. In this context, describing what has been measured differs substantially from deriving predictive insights from omics data and translating them into applicable knowledge. Conceptual advancement depends not merely on technological capacity but on the rigorous interpretation of the information obtained, which ultimately becomes more critical than the technology itself.

In this regard, the integration of such datasets raises a central question: to what extent can biological variables emerging from high-dimensional data be interpreted as evidence of causality? From this concern arises the risk of premature extrapolations, particularly when statistically robust associations are assumed to reflect mechanistic relationships without adequate functional validation. Only through such interpretative depth can a meaningful systems-level perspective of the immune response be achieved, particularly through the integration of multiple omics layers.

Thus, the main challenge is not simply integrating datasets computationally, but ensuring that statistical associations are supported by mechanistic validation and iterative experimental refinement (Svinin and Glaab, 2025).

Overall, no single approach is capable of fully capturing the complexity of the immune system. Although multi-omics integration is highly promising, it also increases the risk of overfitting (McCabe et al., 2020). Furthermore, correlations identified across different technologies do not necessarily imply functional causality. In this context, when multi-omics strategies are applied to rational vaccine design, findings must undergo iterative refinement and rigorous experimental validation to ensure biological and translational robustness (Benabbou et al., 2026).

## 8 Rational vaccine design: from empirical to systems-based approaches

Technological advances have impacted multiple fields, and vaccine development has been no exception. Whereas empirical approaches based on trial and error were previously predominant, the emergence of novel technological tools for vaccine design, together with systems-based approaches, has driven a transition toward the prominence of rational vaccine design (Poland et al., 2011; Schijns et al., 2021; Meyer and Zepp, 2022).

**FIGURE 3:**
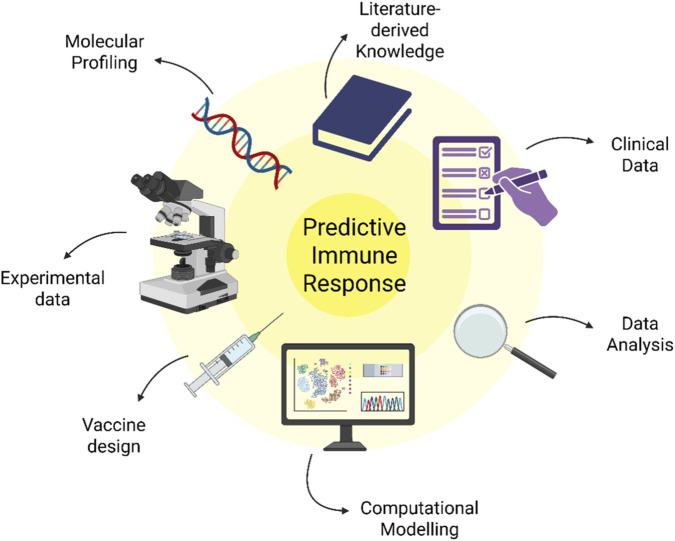
Systems-based framework for predictive immune response in rational vaccine design. The figure illustrates the integration of multiple data sources and analytical processes converging toward the prediction of immune responses. Key components include molecular profiling (omics data), literature-derived knowledge, experimental data, clinical data, computational modeling, and continuous data analysis, all interconnected within an iterative and systems-level approach. Rather than a linear pathway, vaccine development is represented as a dynamic and integrative process supported by multi-dimensional evidence, ultimately guiding vaccine design and optimization (created by the author with BioRender.com).

Within this framework, systems developed to analyze multi-omic data enable several advances, including a deeper understanding of immune responses, predictions of vaccine efficacy, early identification of potential adverse reactions based on biomarkers, discovery of novel immunization-related biological markers, identification of antigens with greater immunostimulatory potential, and the use of improved adjuvants—namely, substances that enhance the immune response (Sharma et al., 2019). It is now possible, for example, to assess the physicochemical properties of particles by considering features such as degradability, shape, size, and surface characteristics, particularly through particle engineering, which underpins these emerging technologies (Gause et al., 2017) (Figure 3).

As illustrated in Figure 3, rational vaccine design emerges from the convergence of multiple analytical and experimental domains, including laboratory experimentation, genomic information, computational modeling, clinical evaluation, and data-driven optimization. Rather than representing a linear pipeline, the figure conceptualizes vaccine development as an iterative and integrative process in which diverse sources of evidence converge toward a targeted immunological objective. This integrative architecture reflects the central argument of this article: that rational vaccine design is grounded in systems-level coordination rather than empirical trial-and-error strategies.

Despite these advances, it is important to acknowledge that technological systems for large-scale data acquisition do not fully capture all properties of the immune system. The immune system is inherently complex and adaptive, capable of generating non-linear and context-dependent responses influenced by factors such as age, microbiota composition, and host genetics (Brodin and Davis, 2017). Therefore, continuous experimental validation remains essential, even within rational design frameworks, to adequately account for the emergent properties that characterize immune responses (Sharma et al., 2019).

In this sense, Figure 3 visually encapsulates the transition from empirical vaccinology to a predictive, model-informed framework supported by systems immunology.

## 9 Adjuvants and immunomodulation from a systems perspective

With the advancement of technology and nanotechnological tools, the development of novel adjuvants using these approaches has become a tangible reality. In this context, the primary objective has been to enhance vaccine efficacy while ensuring the safety of immunized individuals (Jin et al., 2019). However, despite these advances, significant challenges remain in balancing immune potency with tolerability, particularly in diverse patient populations. From a systems vaccinology perspective, adjuvants should not only amplify immune responses but strategically modulate interconnected innate and adaptive pathways to achieve optimal protection without inducing excessive inflammation. From this perspective, adjuvants can be understood not merely as stimulatory agents, but as components capable of reshaping interconnected innate and adaptive pathways.

In this regard, the systems-based perspective of immunity has enabled the development of more effective adjuvants for mucosal vaccines, for example. This is particularly relevant because the mucosal immune system exhibits a predisposition toward tolerance and regulatory responses, which may limit the ability of vaccines to induce robust and protective immunity.

In addition, there is a growing demand for safe and effective immunizers administered via mucosal routes to combat diseases such as AIDS, tuberculosis, influenza, and diarrheal illnesses. Within this framework, emerging technologies have revolutionized the strategies used to address these challenges (Chen et al., 2010).

mRNA vaccines have revolutionized vaccinology by eliciting robust, well-characterized and highly effective immune responses. Their efficacy relies in part on lipid nanoparticles (LNPs), which protect the mRNA and ensure efficient cellular delivery, predominantly via systemic rather than mucosal routes. However, the biodistribution and immunostimulatory properties of LNPs are strongly influenced by physicochemical characteristics such as particle size, surface charge, and PEGylation, which can affect both delivery efficiency and off-target immune activation. Despite this success, LNPs present limitations for inducing mucosal immunity, which is critical for protection against pathogens entering through mucosal surfaces. This limitation highlights a critical opportunity for the rational design of next-generation LNPs or alternative delivery systems that could better target mucosal tissues. In this context, computational modeling and artificial intelligence–guided approaches have emerged as promising tools to optimize LNP design and improve their safety and efficacy profiles (Di Salvatore et al., 2025).

From a systems immunology perspective, integrating multi-omics data could inform the design of next-generation adjuvants and delivery systems that optimize both systemic and mucosal immune responses, tailoring the magnitude, quality, and durability of protection (Sawaran Singh et al., 2025). However, translating these insights into clinically approved vaccines remains a complex challenge due to interindividual variability and regulatory hurdles.

## 10 Challenges and limitations of multi-omics integration in systems vaccinology

Despite the numerous benefits achieved through the integration of omics data, integrating omics data remains a complex task, mainly due to the diversity of technologies employed. One proposed solution to address this limitation is the standardization of terminology across systems, aimed at facilitating automated reasoning through consistent vocabulary definitions within computational tools (He, 2012).

**FIGURE 4:**
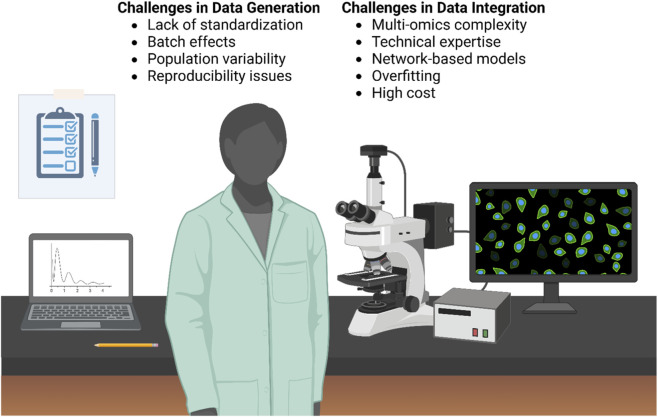
Integrated experimental and computational challenges in multi-omics–driven vaccine research. The figure illustrates the key limitations encountered across the early stages of the multi-omics pipeline, combining experimental data generation and computational integration within a unified laboratory setting. On the left, challenges related to data generation are highlighted, including lack of standardization, batch effects, population variability, and reproducibility issues, which may affect data quality and consistency. On the right, challenges associated with data integration are presented, such as multi-omics complexity, technical expertise requirements, network-based modeling approaches, overfitting, and high cost, all of which can impact analytical robustness and scalability. Together, these interconnected constraints influence the reliability and translational potential of systems-based and AI-driven vaccine development strategies (created by the author with BioRender.com).

The limitations associated with multi-omics integration can be broadly divided into two interconnected domains: (i) sources of biological and methodological variability and (ii) analytical and operational complexity inherent to high-dimensional data integration (Figure 4).

Another important limitation is incomplete coverage and missing data, which necessitate the development of robust strategies to process datasets while accounting for data absence or compromised quality. Furthermore, differences in experimental design, laboratory conditions, reagent batches, technology operators, and other non-biological factors may lead to biased interpretations and erroneous conclusions. Furthermore, each omics technology inherently presents its own technical limitations. When these datasets are integrated in a multidimensional manner, data scales must also be carefully adjusted to produce a unified, interpretable framework that accommodates data heterogeneity.

Moreover, the gap between theoretical data generation and its translation into clinical practice depends, among other factors, on financial investment, infrastructure, and the level of complexity involved in applying these approaches to broader populations (Hemme et al., 2026). Together, these factors represent significant barriers to the translational potential of systems-based approaches. The lack of standardization may hinder direct comparison across studies, generate divergent immune signatures, and lead to inconsistent interpretations.

At both the experimental and population levels, many of these challenges arise from biological variability and methodological heterogeneity. Batch effects, for instance, can artificially suggest biological differences due to technical variability, such as samples processed on different days, handled by different technicians, or generated using distinct reagent lots or sequencing platforms. In addition, the statistical integration of multi-omics data requires sophisticated computational models, technical expertise, and network-based analytical frameworks, which may introduce analytical instability and complicate biological interpretation. Overfitting further limits generalizability, as predictive models developed from high-dimensional datasets with relatively small sample sizes carry a substantial risk of excessive data fitting.

Reproducibility may also be compromised by the aforementioned factors—including methodological heterogeneity, batch effects, population differences, platform variability, and distinct bioinformatic pipelines—thereby impeding the clinical translation of candidate biomarkers and contributing to failures in subsequent validation or clinical trials. Additionally, the high cost of large-scale multi-omics studies limits scalability and complicates implementation, particularly in low-resource settings.

At the analytical and operational level, the growing complexity of multi-omics integration demands advanced computational expertise, robust modeling strategies, and substantial infrastructure, which may further amplify barriers to scalability and implementation.

Finally, global inequity is accentuated by the predominance of data derived from specific populations in high-income countries, restricting immunological diversity in datasets and narrowing the spectrum of diseases investigated. Consequently, endemic diseases in low-income regions may remain underexplored, leading to scientific gaps, limited biomarker generalizability, and translational inequities.

Collectively, these interconnected limitations illustrate how biological variability, analytical complexity, and structural constraints converge to influence the translational potential of systems-based vaccine research, as summarized in Figure 4.

## 11 Clinical and translational implications

Table 1 summarizes key aspects of the interface between bench research and clinical practice, highlighting how findings from systems immunology have influenced vaccine clinical trials, outlining clinical barriers to the implementation of omics-based biomarkers, and presenting illustrative examples of translational successes and challenges.

**TABLE 1:** Applications of Omics Technologies in Licensed Vaccines: Insights into Clinical Impact and Current Limitations. This table summarizes representative examples of licensed vaccines in which omics approaches have contributed to understanding immune responses, clinical outcomes, and current limitations.

Vaccine	Omics approach	Clinical impact	Limitation	Reference
BNT162b2 (mRNA Covid Vaccine)	Transcriptomics	Following booster vaccination, an approximately 100-fold increase in the frequency of a myeloid cell cluster enriched in interferon-response transcription factors and depleted in AP-1 transcription factors was observed. This finding enabled the identification of distinct innate immune pathways associated with CD8^+^ T cell responses and neutralizing antibody production, allowing the researchers to demonstrate a correlation between the monocyte transcriptional signature and neutralizing antibody responses against the B.1.351 variant	A robust characterization of adaptive immunity induced by BNT162b2 has been established; however, comparatively little is known about the innate immune responses elicited by this or other mRNA vaccines	Arunachalam et al. (2021)
RTS,S (Malaria)	Transcriptomics	Overall results from multiple transcriptomic studies in malaria-naïve adults: central role of major histocompatibility complex class I, NF-κB, and interferon signaling pathways in the induction of RTS,S-mediated protection against controlled human malaria infection	Few studies have been published on the use of transcriptomics for malaria vaccines other than RTS,S, and only a limited number of investigations have analyzed RTS,S-induced protection in populations with prior naturally acquired malaria exposure	Stanisic and McCall (2021)
4CMenB (Meningococcus B)	Genomics	Identification of 28 novel protein antigens capable of eliciting bactericidal antibodies, together with the immunogenicity and safety demonstrated in clinical trials in infants, children, adolescents, and adults	One clinical study reported an adverse reaction, namely, high rates of fever, in children who received the antigenic lipoprotein rLP2086 at doses of 20 or 60 μg, whereas the remaining studies demonstrated favorable tolerability, safety, and efficacy outcomes	Gasparini et al. (2015)

## 12 Ethical regulatory and data governance considerations

The increasing reliance on multi-omics integration, predictive modeling, and AI-driven analytics in systems vaccinology extends the discussion beyond technical performance into ethical, regulatory, and governance domains. In this context, the ethical and legal dimensions associated with these advances must be carefully addressed.

AI in healthcare enables more accurate diagnoses, personalized treatments, and other innovations that underpin precision medicine. Moreover, it holds significant potential for early disease detection, clinical decision support, and the discovery and development of novel therapeutics. Nevertheless, the implementation of AI in the healthcare sector is accompanied by a complex network of ethical challenges (Li et al., 2024; Mohsin Khan et al., 2025). As predictive models become increasingly central to rational vaccine design and immune response modeling, concerns regarding transparency, interpretability, and accountability become inseparable from their technical implementation (Kumar et al., 2024; Niu et al., 2025).

Data privacy and protection are fundamental to preserving patient trust and ensuring data integrity. The collection, storage, and use of health-related data must be conducted with rigorous safeguards, including anonymization procedures, encryption methods, and secure data-sharing protocols. Ensuring fairness and transparency in AI deployment represents another critical challenge. Patients should be clearly informed about the use of AI-based systems, understand how these technologies operate, and retain autonomy in deciding whether to consent to their application.

Accountability also remains a major concern. In the event of errors or adverse outcomes associated with AI systems, clear mechanisms must be established to determine responsibility. Therefore, the development and implementation of structured, accessible, and standardized ethical guidelines and regulatory frameworks are essential to ensure the safe, equitable, and sustainable integration of these technologies into clinical practice (Elendu et al., 2023).

From a governance perspective, big data continues to pose significant challenges due to the absence of standardized global regulation. Divergent and sometimes conflicting national and international legal frameworks create barriers to the harmonized advancement and responsible use of data-driven technologies. This fragmentation complicates cross-border data sharing, limits large-scale collaborative research, and hinders the development of interoperable infrastructures. Consequently, establishing coherent and internationally aligned governance frameworks is essential to foster innovation while ensuring ethical oversight, legal clarity, and the protection of individual rights (Goirand et al., 2021).

In this context, the approval of data-driven vaccines is far from straightforward. This complexity reflects not only traditional regulatory requirements but also the need to evaluate algorithmic stability, dataset representativeness, and reproducibility across heterogeneous populations. Although such vaccines may demonstrate safety and efficacy during development, regulatory authorization requires rigorous verification of multiple dimensions, including algorithmic integrity, data monitoring systems, and adherence to good practices across laboratory research, manufacturing processes, and clinical investigation. Regulatory agencies play a central role in these evaluations, ensuring both technical robustness and public safety.

The approval pathway is often lengthy and may vary substantially across countries, depending on national regulatory frameworks and available procedures. Differences in evidentiary standards, review timelines, and post-marketing requirements further contribute to this heterogeneity. Regulatory decisions are ultimately grounded in comprehensive scientific and epidemiological evidence. Importantly, oversight does not cease upon licensure; continuous post-marketing surveillance is essential to monitor safety and effectiveness and to ensure that no deviations occur that could compromise public health (Nalin, 2002).

## 13 Artificial intelligence and machine learning in systems vaccinology

Within this integrative framework, artificial intelligence functions not as an isolated technological tool, but as a computational extension of multi-omics data integration. Its ability to learn from data through repeated exposure, combined with substantial computational power and the capacity to process large-scale datasets, has introduced a new paradigm in data analysis that is less reliant on prior assumptions. Within AI, machine learning (ML) constitutes a major subfield, which in turn encompasses deep learning (DL). The distinction between these approaches lies primarily in their architecture and feature-learning processes. Deep learning is based on multilayered artificial neural networks capable of automatically learning complex features and generating informative embeddings directly from raw data, without requiring manual feature engineering. In contrast, classical machine learning methods typically depend on predefined features. For example, when distinguishing among images containing different types of flowers, conventional ML algorithms require prior specification of measurable attributes—such as length and width—to enable the model to discriminate between classes.

Translating these concepts to biological applications in omics sciences, artificial intelligence has emerged as a powerful tool for analyzing complex biological systems. The proteome, for instance, represents an exceptionally complex system that, unlike the genome, cannot be amplified, thereby posing significant analytical challenges. AI-based methodologies may help address these limitations by enhancing pattern recognition and enabling integrative analysis of high-dimensional proteomic data (Narayanan et al., 2025; McKenzie et al., 2026). Artificial intelligence systems, when applied to immunology, can construct predictive frameworks for immune responses, encompassing both intuitive and counterintuitive patterns. By leveraging high-dimensional immunological data, these models can reveal complex interactions that may not be readily apparent through conventional analytical approaches.

Concerning the application of machine learning to multi-omics data, several key statistical challenges must be emphasized, including high dimensionality, multicollinearity, heterogeneity, and sparsity. These intrinsic properties significantly affect model performance, generalizability, and interpretability. In this context, integrative analytical frameworks typically involve structured data preprocessing pipelines followed by coordinated computational steps. Broadly, these approaches aim to identify associations across distinct omics layers that converge into a shared latent subspace, thereby enabling coherent biological interpretation. To uncover such common representational spaces, statistical methodologies are frequently combined with concepts derived from dictionary learning and other dimensionality reduction techniques. These strategies facilitate the extraction of structured patterns and latent factors that capture shared biological signals while mitigating noise and redundancy across heterogeneous multi-omics datasets (Xiao et al., 2023).

Despite these advances, several limitations must be considered. One of the most critical is overfitting, particularly in high-dimensional datasets with relatively small sample sizes, which can compromise model generalizability. In addition, the complexity of deep learning models often results in reduced interpretability. While simpler models may offer more transparent and intuitive insights, highly complex architectures can behave as “black boxes,” limiting the ability to extract mechanistic understanding from predictions. Another important limitation relates to data dependency. Machine learning models require large, high-quality datasets with comprehensive feature representation to achieve robust performance. Critical variables—such as age, comorbidities, genetic background, and treatment exposure—must be adequately captured to ensure accurate predictions. Insufficient or biased data may compromise model validity and amplify systematic errors. Furthermore, models trained on restricted geographic or genetic populations may inadvertently reinforce disparities in vaccine response prediction, safety assessment, and clinical applicability across underrepresented groups.

These limitations raise important ethical and clinical concerns, particularly when AI systems contribute to healthcare decision-making. Reduced interpretability may hinder clinicians’ ability to justify model-derived recommendations, potentially affecting trust and accountability (Mann et al., 2021). Moreover, predictive accuracy does not necessarily imply mechanistic understanding, and correlations identified by computational models require experimental validation to ensure biological plausibility. Excessive reliance on AI without empirical validation may therefore lead to misleading or clinically inappropriate conclusions. For this reason, artificial intelligence should not be regarded as an autonomous decision-making tool, but rather as a complementary approach embedded within a framework of rigorous scientific oversight. Human supervision remains essential to ensure safety, interpretability, and ethical responsibility in AI-driven immunological research and applications (Elfatimi et al., 2025).

Despite these challenges, emerging evidence highlights the potential of artificial intelligence in areas such as pharmacovigilance. Machine learning approaches have demonstrated utility in improving the detection and monitoring of adverse events, enabling earlier signal identification and more efficient analysis of large and heterogeneous datasets (Kim et al., 2021). When appropriately validated and integrated with clinical expertise, these tools may significantly enhance data-driven safety surveillance.

## 14 Populational diversity and access

Systems vaccinology contributes to a deeper understanding of immune responses, which are influenced by genetic background, health status, prior exposures, environmental factors, the microbiome, among other determinants (Dib et al., 2024).

Within this context, it becomes imperative to critically examine the population sampling strategies currently employed in clinical trials for vaccine development based on omics technologies. Questions naturally arise, such as: “Is genetic diversity being adequately captured by these methodologies?”, “Are all countries appropriately represented?”, “Do the diseases under investigation include those most prevalent in lower-income countries?”, and “Is there any form of global equity in this process?”.

For instance, in the case of malaria, particularly among populations within the endemic belt—Sub-Saharan Africa, parts of South Asia, and South America—vaccine design would ideally incorporate multiple epitopes informed by population immunoinformatics analyses. However, access to the tools required to implement such technologies remains largely restricted to higher-income countries, thereby concentrating the benefits of computational vaccinology within these regions (Hashim and Dimier-Poisson, 2025).

Importantly, merely establishing mRNA vaccine production facilities does not ensure successful implementation. Additional factors must be considered, including the availability of raw materials required for mRNA production, logistical complexity across the manufacturing supply chain, associated costs, capacity building and workforce training, intellectual property constraints, compliance with internationally approved standards, and the need to address local vaccine hesitancy.

In this regard, the World Health Organization (WHO) has undertaken initiatives aimed at reducing inequalities related to these challenges (Kairuz et al., 2022).

In summary, there remains a clear underrepresentation of populations from the Global South, and significant challenges persist in achieving global health equity. Nevertheless, initiatives such as those led by the WHO have been implemented and are yielding positive progress toward this goal.

## 15 Empirical vs. systems-based approaches

The key differences between empirical and systems-based vaccinology are summarized in Table 2, highlighting shifts in study focus, methodological approaches, and data integration strategies.

**TABLE 2:** Comparison between empirical and systems-based approaches in vaccinology. This table contrasts key features of empirical and systems-based vaccinology, including study focus, methodological strategies, resource requirements, and analytical scope.

Aspect	Empirical vaccinology	Systems-based vaccinology	Reference
Study foccus	Pathogen-centered	Multi-layered (transcriptome, proteome, metabolome, epigenome, interactome, phosphoproteome, glycome, lipidome, etc.)	Ponne et al. (2024)
Method	Trial-and-error approach	Bioinformatics and computational analysis	
Pathogen culture requirement	Required	Not necessarily required	
Time required	Longer	Shorter*	
Types of proteins considered	Structural	Structural and non-structural proteins	

* requires experimental validation.

## 16 Future perspectives

Although numerous vaccines are available for a wide range of diseases, many immunizations still require improvement, mainly due to the substantial resistance exhibited by certain pathogens.

In this context, representative examples include the human immunodeficiency virus (HIV), respiratory syncytial virus (RSV), *Bordetella* pertussis, influenza virus, *Mycobacterium tuberculosis*, and Plasmodium species responsible for malaria, as well as pathogens with the potential for future emergence of variants, such as those associated with severe acute respiratory syndrome (SARS) and Ebola virus disease.

In this scenario, systems immunology grounded in multi-omics data and applied to synthetic immunology—particularly in vaccine development—emerges as a transformative approach to how infectious diseases can be prevented and controlled (Raeven et al., 2019).

Looking ahead, the development of personalized vaccines is a feasible prospect, as it is already known, for example, that influenza vaccines administered to adults aged 65 years and older are more effective when they contain four times more antigen than standard influenza vaccines. Consequently, the potential impact on public health is substantial, as novel vaccination strategies and rational vaccine administration approaches can be developed (Sugrue and Duffy, 2024).

## 17 Conclusion

This article has argued that systems immunology, supported by multi-omics integration and computational modeling, constitutes not merely a technological advancement but a structural transformation in vaccine science. By moving beyond reductionist and predominantly empirical paradigms, systems vaccinology enables a mechanistically informed and predictive framework for rational vaccine design.

The integration of distinct biological layers offers unprecedented opportunities to understand immune complexity; however, this potential can only be realized if technological capacity is accompanied by rigorous biological interpretability, methodological standardization, and experimental validation. Without such interpretative depth, high-dimensional data risk amplifying noise rather than generating actionable insight.

Therefore, systems vaccinology should not be understood as a simple extension of empirical approaches, but as a paradigmatic reorientation of how immunity is conceptualized, modeled, and therapeutically modulated. Its future development depends on the responsible integration of artificial intelligence, robust data governance, and globally representative datasets that prevent the reinforcement of biological and geopolitical inequities.

Ultimately, the transition toward rational, data-driven vaccine design is not optional in the face of emerging pathogens, population heterogeneity, and complex immunological landscapes. It represents a necessary evolution in vaccinology — one that must be scientifically rigorous, ethically grounded, and globally inclusive to fulfill its promise for public health.
