# Supplementary material for: From empirical vaccinology to predictive systems-based vaccine design: multi-omics integration, artificial intelligence, and global equity challenges
Source: Front Syst Biol. 2026 Apr 30;6:1819469. doi: 10.3389/fsysb.2026.1819469 (PMC13171360; doi:10.3389/fsysb.2026.1819469)
Supplement: Supplementary file 1 [file Supplementaryfile1.docx]

Prompts

- "Com base nas informações do site do Research Topic, monte um roteiro de publicação com possíveis temas para artigo de revisão."
- "Refaça o roteiro considerando o prazo como final de fevereiro."
- "Descreva o roteiro como um passo a passo e sugira o melhor tema."
- "Monte um roteiro final bem detalhado com tudo que preciso fazer, saber e procurar para entregar até o fim de fevereiro, garantindo tempo hábil antes da editora avaliar”
- “Traduza para inglês formal de artigo científico”
- “Traduza para inglês formal de revista internacional”
- “Traduza para português”
- “Opine”
- “Detalhe mais o que eu preciso fazer em cada seção”
- “Aponte detalhadamente o que preciso mudar”
- “Faça um roteiro em tópicos para que eu possa discorrer sobre eles com minhas palavras”
- “Como deixar no nível de Frontiers?”
- “Como reduzir didatismo?”
- “Como tornar mais crítico?”
- “Como deixar mais autoral?”
- “Sugira o melhor título possível”
- “Como fortalecer conclusão?”
- “Como integrar seções (IA, ética, equidade)?”
- “Como estruturar abstract ideal?”
- “Faça um resumo em tópicos bem sucinto”
- “Onde eu devo realizar essas alterações?”
